# Estradiol prevents olfactory dysfunction induced by A-β 25–35 injection in hippocampus

**DOI:** 10.1186/1471-2202-14-104

**Published:** 2013-09-24

**Authors:** Carlos Bernal-Mondragón, Selva Rivas-Arancibia, Keith M Kendrick, Rosalinda Guevara-Guzmán

**Affiliations:** 1 Departamento de Fisiología, Facultad de Medicina, Universidad Nacional Autónoma de México. Apdo, Postal 70250, D.F. México, Delegación Coyoacán 04510, Mexico; 2 Key Laboratory for Neuroinformation, School of Life Science & Technology, University of Electronic Science & Technology of China (UESTC), 610054, Chengdu, P.R. China

**Keywords:** Amyloid beta, Neurodegeneration, Neuroprotection, Estrogen, Alzheimer’s disease, Olfactory disfunction

## Abstract

**Background:**

Some neurodegenerative diseases, such as Alzheimer and Parkinson, present an olfactory impairment in early stages, and sometimes even before the clinical symptoms begin. In this study, we assess the role of CA1 hippocampus (structure highly affected in Alzheimer disease) subfield in the rats’ olfactory behavior, and the neuroprotective effect of 17 beta estradiol (E_2_) against the oxidative stress produced by the injection of amyloid beta 25–35.

**Results:**

162 Wistar rats were ovariectomized and two weeks after injected with 2 μl of amyloid beta 25–35 (A-β_25–35_) in CA1 subfield. Olfactory behavior was evaluated with a social recognition test, odor discrimination, and search tests. Oxidative stress was evaluated with FOX assay and Western Blot against 4-HNE, Fluoro Jade staining was made to quantify degenerated neurons; all these evaluations were performed 24 h, 8 or 15 days after A-β_25–35_ injection. Three additional groups treated with 17 beta estradiol (E_2_) were also evaluated. The injection of A-β_25–35_ produced an olfactory impairment 24 h and 8 days after, whereas a partial recovery of the olfactory behavior was observed at 15 days. A complete prevention of the olfactory impairment was observed with the administration of E_2_ two weeks before the amyloid injection (A-β_25–35_ 24 h + E_2_) and one or two weeks after (groups 8 A-β +E_2_ and 15 A-β +E_2_ days, respectively); a decrease of the oxidative stress and neurodegeneration were also observed.

**Conclusions:**

Our finding shows that CA1 hippocampus subfield plays an important role in the olfactory behavior of the rat. The oxidative stress generated by the administration of A-β_25–35_ is enough to produce an olfactory impairment. This can be prevented with the administration of E_2_ before and after amyloid injection. This suggests a possible therapeutic use of estradiol in Alzheimer’s disease.

## Background

Alzheimer’s disease (AD), the most common neurodegenerative disorder in humans, is characterized by deterioration of cognitive and mental functions, including learning and memory skills; particularly those involving medial temporal lobe regions, such as the hippocampus [1]. Interestingly, odor perception and learning, which also involve medial temporal lobe structures, are often impaired early in the course of AD, and therefore olfactory processing deficits may be a clinical manifestation of early pathology [2-4]. Studies have generally reported olfactory discrimination and learning deficits early in AD [5,6] followed by additional problems in detecting odors as the disease progresses [5,7]. There are many studies reporting the relation of olfaction impairment with different neurodegenerative diseases such as Parkinson, Alzheimer and Huntington. Devanand [8] reported that individuals who had presented olfactory dysfunction in the UPSIT test, two years later developed AD. The relationship between olfactory impairment and cognitive deficit in some neurodegenerative diseases such as Alzheimer has been well described, but the underlying mechanism of this relationship is unclear [9]. It has been shown that AD is characterized by the formation of extracellular deposits of A-β peptide [10] leading to the formation of neuritic plaques, neurofibrillary and intraneuronal tangles of hiperphosphorylated *tau* protein, as well as by the microglia activation [11] in cortex and hippocampus.

It has been reported that one of the action mechanisms of A-β is through oxidative stress [12,13]. Several authors have used the A-β_1–42_ peptide in animal models to study AD. However, the fragment 25–35 of A-β seems to be the neurotoxic part of the whole protein. This fragment is capable of producing oxygen species that lead to neurodegeneration by oxidative stress production only [12,14]. In hippocampus, the injection in CA1 results in a neuronal degeneration and cell loss of the pyramidal cell layer affecting spatial memory in rats [15].

A-β_25–35_ cannot be produced through typical APP processing, but it is often selected as an alternative model to full-length A-β because it retains both its physical and biological properties. Perhaps the most important factor which was found to influence toxicity, however, was the aggregation state forming fibrils with β-structure and retaining the toxicity of the full-length peptide [16-19]. A-β_25–35_, though not present in humans, is widely used by researchers instead of endogenous fragment A-β_1–42_, which is not found to be at least as toxic as the full-length fragment [14,20].

The first reports on *in vivo* A-β_25–35_ were from a series of studies made by Maurice 1996 [21] and Delobette in 1997 [22] who demonstrated amnesia in mice and rats injected with this fragment. Likewise, long term or single A-β_25–35_ i.c.v injection induced a decline in social recognition behavior in rats [15,23,24] as well as impaired learning in a water maze test [21,22,25] and working memory in a Y maze or radial arm maze [15,21,26-28].

A useful animal model for investigating effects of A-β protein in AD has been to inject different versions of it directly into the brain. Thus, we are using in this project the A-β_25–35_ fragment in an olfactory behavior paradigm. To the best of our knowledge, no studies have so far investigated the effects of this fragment on olfactory perception and memory.

Estrogen is thought to play a protective role against neurodegeneration through a variety of mechanisms and to influence cognitive processes such as learning and memory. The mechanisms implicated include the activation of growth factors, the control of synaptic plasticity and reduced effects of toxicity [29]. There is some evidence to suggest that exposure to estrogen decreases the risk and delays the onset and progression of AD, most probably by reducing A-β production [30,31]. It has also been reported E_2_ inhibits generation of superoxide radicals, thus preventing further propagation of reactive oxygen species (ROS) [32]. It has also been shown to interfere both with A-β production and clearance *in vitro* and *in vivo* in murine models [33].

In the present study, we have therefore investigated first whether A-β_25–35_ injected directly into the hippocampus (HIPP) or into the olfactory bulb (OB) in ovariectomized female rats produced both neurodegenerative changes in these regions and impaired olfactory perception and learning as well as spatial memory (spontaneous alternation). And secondly, whether treating animals with E_2_ can prevent some or all of these effects.

## Methods

### Subjects

Adult female Wistar rats were used in the study. They were group-housed (4–5 per cage) with food and water available *ad libitum* and with an artificial 12 h light/dark regime (lights were on from 7 am to 7 pm). All experiments and animal welfare conditions were approved by the Ethical Committee of the Faculty of Medicine at the Universidad Nacional Autónoma de México and in accordance with the European Communities Council Directive. All efforts were made to minimize the number and suffering of animals used.

A total of 162 adult virgin female three-month Wistar rats from our house breeding colony were used as subjects and further 63 (20–22 days old) juvenile animals were used as test *stimuli* in the social recognition task. Adults weighed 248.42 g ±12.6 g and juveniles 112 ± 6.48 g. The adult animals were ovariectomized under general anesthesia (ketamine/xylazine mixture, 15 mg/kg + 1 mg/kg, i.p) 15 days prior to the experimental procedure. In order to minimize the number of juvenile animals used, they were rotated for control and experimental groups. Adult and juvenile animals were caged individually 1 h prior to the social recognition tests and during the 60 min inter exposure interval. All the experiments were conducted during the light phase of the cycle, between 0700 h and 1300 h.

### Injection of A-β

**Figure 1:**
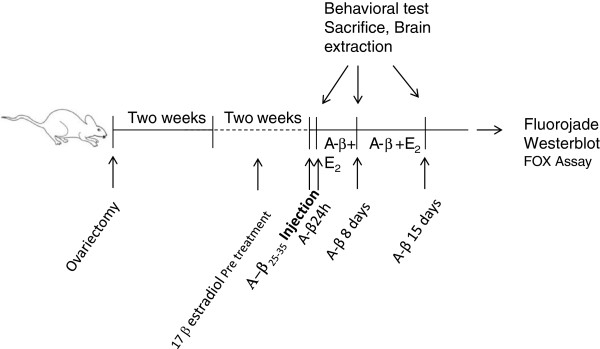
**Scheme of time line of the procedure used.** All rats were ovariectomized and let them recover for two weeks before any procedure was made. All experimental groups were pre-treated with 17 beta estradiol (E_2_) (25 μg/kg) for two weeks before A-β_25–35_ injection in HIPP or OB. 8 or 15 days + E_2_ groups received one or two weeks respectively additional injections of E_2_. Olfactory tests and brain extraction were carried out 24 h, 8 or 15 days after A-β_25–35_ injection.

Stereotaxic surgery: All ovariectomized adult female Wistar rats were anesthetized with a ketamine/xylazine mixture (15 mg/kg + 1 mg/kg, i.p) and stereotaxic surgery was performed in a standard rodent stereotaxic frame (David Kopf, USA). The animals were divided into six control and twelve experimental groups (n = 9 animals per group) for the stereotaxic surgery. Three control groups were assigned for bilateral injection of phosphate buffer solution in HIPP and three more for OB, tested 24 h, 8 and 15 days after vehicle injection for social recognition behavior. Three experimental groups were injected with 2 μl of A-β_25–35_ (1 μg/100 μM) dissolved in phosphate buffer and previously incubated at 37°C in a shaking-water bath for 72 h (to induce aggregation state) into the HIPP, tested 24 h, 8 or 15 days after A-β_25–35_ injection and other three groups were injected before and after A-β injections with E_2_, 25 μg/kg s.c. daily injections for two weeks or one or two additional weeks (groups evaluated 8 days + E_2_ or 15 days + E_2_ after A- β_25–35_ injections respectively) and using propylene glycol as a vehicle. As a control for the E_2_ injections, three additional groups received daily injections with the vehicle (subcutaneously propylene glycol) for two weeks and afterwards for one or two weeks (same as described in upper paragraph for E_2_ injections) (See Figure 1). Co-ordinates for HIPP A-β_25-35_ and control injections were 4.2 mm posterior to Bregma, 3.0 mm lateral from midline and 2.6 mm ventral to dura [34]. Another six experimental groups received a bilateral injection of the A-β in the OB’s coordinates: 7.1 mm rostral to bregma, 1.5 mm lateral to the midline, and 1.5 mm ventral to dura [34]. Three of these groups also received E_2_ for two weeks and one or two weeks after, prior to A-β injection and tested either 24 h, 8 or 15 days later (same as HIPP groups); other groups received vehicle injections for two weeks and one or two weeks after A-β_25–35_ injection. After recovery from surgery, animals were housed together in groups. After behavioral testing was completed, the animals were sacrificed by decapitation and their brains removed and stored at -80°C for subsequent analysis.

### Social recognition memory test

The social recognition procedure was similar to that described in our previous papers [35,36]. The protocol used was as follows: starting two days prior to A-β or control vehicle injection in HIPP or OB and just before the test, each adult rat was habituated to the test cage daily for four minutes (50X50X42 cm). Each testing session consisted of a sequence of three 4-min trials. The first trial for the adult rat was a habituation period to the test cage; the second trial was the first encounter between the adult rat and the juvenile rat (social memory acquisition); the third trial was a re-exposure to the familiar animal together with an unfamiliar juvenile *stimulus* animals introduced simultaneously into the test cage 60 min after the social memory acquisition trial (IEI). Experimental groups were tested 24 h, 8 and 15 days after A-β_25–35_ injection into either HIPP or OB with or without E_2_ treatment. Following each test, the cage was thoroughly cleaned. Video recording of investigatory behavior was used to assess the time spent by adult rats investigating the *stimulus* animal in the social recognition test. The data collected from video-recordings were transferred to an IBM computer for off-line analysis. Behaviors considered related to social recognition learning and memory were anogenital sniffing, close following, and pawing of the *stimulus* animal. The percentage of time investigating the familiar compared to that with the unfamiliar one was measured [37]. A selective recognition memory was considered present if there was first, a significant reduction in the mean duration time of exploration, between the first two encounters with the *stimulus* juvenile; and, secondly, if there was also significantly greater investigation time of the novel juvenile in the third trial compared with that for the familiar juvenile.

### Olfactory perception and habituation tests

To test for possible general olfactory perception impairments, additional groups of ovariectomized animals were used (n = 9/group); for both HIPP and OB injections, one group was injected with vehicle alone and tested after 24 h, other groups were A-β_25–35_ injected and tested at 24 h, 8 and 15 days later (independent groups). Another group was pre-treated with E_2_ for two weeks prior to A-β injection (A-β_25–35_ + E_2_, 24 h group). Two additional groups were injected additionally with E_2_ for one or two weeks, after A-β_25–35_ injection (A-β_25–35_ + E_2,_ 8 days and A-β_25–35_ + E_2,_ 15 day groups). For the olfactory perception test, individual animals were temporarily transferred from their home cage to another acrylic box and placed in the center, while a small piece of chocolate was buried in a random corner in the bedding of their home cage. Each animal was tested once and then returned to its home cage. The time it took them to locate and eat the chocolate chip was recorded (latency (up to a maximum of 120 s)). Latency to locate the buried piece of chocolate was the dependent variable in this analysis.

After the social recognition test was completed, we also evaluated non-social odor discrimination skills in all groups (adapted from Paolini and McKenzie) [38]. A lemon scented filter paper was introduced into a small perforated tube (5 cm long, 1.5 cm diameter) which was fixed on one of the walls of the experimental cage and the animal was allowed to explore it for two minutes. We repeated this procedure three times with ten-minute intervals between trials (IEI) and with the same scent (lemon) (three habituation trials). In the fourth discrimination trial, a vanilla scent was poured to the filter paper and the procedure was repeated. In order to test an odor preference, we also used other scents such as coffee and orange. We did not observe any preference or aversion to these odors (data not shown).

### Spontaneous alternation behavior in a T-maze

A T-maze test [39] has been widely used to assess spatial memory in rats. This test analyzes the natural spontaneous exploratory behavior of rodents and other species [40]. We used this test to evaluate effects of A-β_25–35_ injection into the HIPP. Same control and experimental groups tested in the social recognition memory detailed above were used to evaluate effects on spatial memory. The T maze was made of black painted wood and covered by clear Plexiglas. Each arm was 30 cm long, 12 cm wide and 10 cm high. The floor of each arm was covered with paper, which was changed between trials. Each rat was placed at the end of one arm and allowed to move freely through the maze for eight minutes. The number of arm entries made by the animals, including returns into the same arm (errors), was visually recorded. Alternation was defined as entries into all three arms on consecutive occasions (triplets).

### Measurement of lipid peroxidation (LPO)

After behavioral tests, control and experimental animals were sacrificed, their brains were placed on an ice-cold plate and HIPP, OB and frontal cortex dissected out and weighed immediately after. Each structure was homogenized in PBS 1:20 and divided into two tubes which were stored at −80°C until the day of the assay for LPO using a FOX assay Kit or for Western Blot. LPO was measured using the Peroxidetect kit (Sigma-Aldrich) which measures the colored adduct formed by xylenol orange and Fe_3_+ generated in presence of peroxides. Sample lipids were extracted using the Bligt & Dyer Protocol. For each ml of sample, 3.75 ml 1:2 (v/v) of CHCl_3_: MeOH was added and mixed. In a second step, 1.25 ml of CHCl_3_ was added and mixed, and then 1.25 ml of dH_2_O was added and mixed. The samples were centrifuged at 1000 RPM for five minutes at room temperature to obtain a two-phase system and from which the organic phase was recovered. 100 μl of the sample was placed in a tube; 1 ml of the working color reagent prepared from the kit was added. The mixture was incubated for 30 minutes at 25°C: the samples were read in a spectrophotometer at 560 nm using methanol as blank. A standard curve of t-BuOOH was plotted. Nanomols of peroxide were calculated using the standard curve and according to the formula:

$$\begin{array}{l} \text{LPO value in nmol}/\text{ml}\;=\;\left(\text{Es}‒\text{Eb}\right)X50.0/\left(\text{Estd}\right)\\ \;X\;\left(\text{Sample}\;\text{volume}\right)\;(\text{Es}\;=\;\text{Sample Absorbance},\\ \;\text{std}\;=\;\text{Absorbance of}\;1\;\text{nmol}/\text{peroxide from}\\ \;\text{the standard curve},\;\text{Eb}\;=\;\mathrm{Blank absorbance}). \end{array}$$

### Western blot for 4-hydroxinonenal

A Western Blot assay for quantifying 4-hydroxinonenal (4-HNE) adduct levels was performed. Proteins were separated by sodium dodecyl sulfate polyacrylamide gel electrophoresis (SDS-PAGE 10%) and transferred to nitrocellulose membranes. Membranes were collected and dried at room temperature until used. The membranes containing the samples (OB or HIPP) were blocked with 5% skimmed milk in TBS-T = 0.01% of Tween 20 (TBS-T) for 2 h at 37°C, and incubated with anti 4-HNE (R&D Systems) (1:1000) overnight under gentle shaking at 4°C. Membranes were rinsed three times with TBS-T, and thereafter were incubated with goat anti-rabbit IgG conjugated with horseradish peroxidase (1:10,000) (Sta. Cruz) for 1 h followed by three times rinsing with TBS-T. Recognized bands were visualized by chemiluminiscence (ECL, General Electric).

### Fluoro-Jade Staining

Degenerating neurons in HIPP and OB were labeled using Fluoro-Jade staining. All labeled neurons from the dorsal hippocampus were counted. Four sections from each brain were used for statistics. For this technique, brains were first embedded in paraffin, cut into 7 μm sections using a microtome and mounted on glass slides. Slides were then first immersed in a solution containing 1% sodium hydroxide in 80% alcohol (20 mL of 5% NaOH added to 80 mL absolute alcohol) for five minutes. This was followed by two minutes in 70% alcohol and two more minutes in distilled water. The slides were then transferred to a solution of 0.06% potassium permanganate for 10 minutes on a shaker table to ensure consistent back ground suppression between sections. They were then rinsed in distilled water for two minutes. The staining solution was prepared from a 0.01% stock solution for Fluoro-Jade C that was made by adding 10 mg of the dye powder to 100 mL of distilled water. To make up 100 mL of staining solution, 4 mL of the stock solution was added to 96 mL of 0.1% acetic acid vehicle. After 20 minutes in the staining solution, the slides were rinsed for one minute in each of three distilled water washes. Excess water was removed by briefly (about 15 s) draining the slides vertically on a paper towel. The slides were then placed on a slide warmer set at approximately 50°C, until they were completely dry. The dried slides were cleared by immersion in xylene for at least a minute before the analysis. For analysis, the average numbers of stained cells were counted in four sections from the HIPP and OB of each animal. The sections were taken from the coordinates mentioned above.

### Statistics

Behavioral data obtained during the social recognition task were expressed as ratios (investigation times of unfamiliar (unfamiliar + familiar)). Because ratios violate the homogeneity of variance assumption required by parametric statistics, the duration of social investigation ratios were arcsine-transformed prior to analysis [arcsin $\left(\sqrt{\mathrm{ratio}}\right)$] [37]. Social investigation times were recorded for each animal and then these values were averaged and transformed according to the experimental group. To test for an overall effect of treatment (A-β_25–35_ injection) on the exploration time, a three factor analysis of variance (ANOVA) was carried out with brain region, treatment and treatment duration as factors reaching a significant difference of p < 0.05; post hoc planned contrast comparisons (corrected for multiple comparisons *Tukey* test) were made using a SPSS 15.0. Effects of A-β_25-35_ on discrimination of different odors were tested using a 4-way ANOVA with brain region (HIPPO/OB), treatment (vehicle, A-β_25–35_, E2), treatment duration (24 h, 8 and 15 days) and odor trials as factors and followed by post-hoc tests. Effects of A-β_25-35_ on latencies to locate buried chocolate, LPO and Fluoro-Jade staining were also analyzed using two or three-way ANOVAs. The results showed a significant statistical difference of p < 0.05 followed by *Tukey* post hoc tests.

## Results

**Figure 2:**
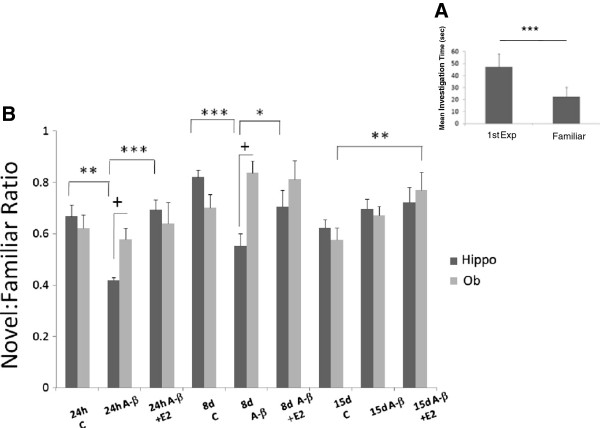
**Olfactory memory acquisition. A**. Olfactory memory acquisition in social investigation, control group. **B**. Ratios of time investigating the familiar or the unfamiliar juvenile rat. Adult ovariectomized female rats tested at 60 min IEI. Control groups (vehicle) evaluated at 24 h, 8 and 15 days after vehicle injection. Experimental groups injected with A-β_25-35_ and A-β +E_2_. HIPP (black columns) or OB (grey columns) evaluated at same times as control groups. *P < 0.05; **P < 0.01; ***P < 0.001, comparison intergroup. +P < 0.05 comparison between OB and HIPP groups.

Figure 2 shows that injection of A-β_25–35_ in hippocampus decrease the novel:familiar ratio, increasing the investigation time for the familiar juvenile in the second encounter; in the groups evaluated 24 h and 8 days after injection, the animal is unable to distinguish between the juvenile familiar from the juvenile unfamiliar odor. The administration of E_2_ reestablishes the time investigation as control groups. No effect was observed if the A-β_25–35_ injection was applied in the olfactory bulb. A three way ANOVA revealed main effects of treatment (Vehicle, Amyloid beta or E2) (F_2,161_ = 5.64, p = 0.004) and treatment duration (24 h, 8 or 15 days) (F_2, 161_ = 10.771, p < 0.001) but not at brain region (HIPP or OB) (F_1,161_ = 0.805, p = 0.371). There were also significant interactions between treatment and treatment duration (F_4,161_ = 3.717, p = 0.007) as well as treatment and brain region (F_2, 161_ = 6.574, p = 0.002) but not between treatment duration and brain region (F_2, 161_ = 2.499, p = 0.086). These show that treatment effects occurred mainly in the HIPP, which were also reduced over time in the HIPP. Finally, there was also a treatment x treatment duration x brain region interaction (F_3, 161_ = 2.996, p = 0.033) indicating again that treatment and treatment duration effects mainly occurred in the HIPP rather than in OB. Post-hoc comparisons revealed there were significant differences between HIPP and OB in both 24 h (p = 0.03) and 8 day (p < 0.001) groups, but no differences were found between the 15 day treatment groups (p = 0.542, NS). Post-hoc tests revealed that social recognition memory was significantly impaired by A-β injection in HIPP in both the 24 h (p < 0.001) and 8 day (p < 0.001) treatment groups compared with that of control groups (vehicle). No differences were found with the 15 day treatment group compared with that of the control group (F5, 54 = 4.30 p = 0.73, NS). Pre-treatment with E2 in the HIPP groups significantly improved olfactory recognition memory in the 24 h (p < 0.001) and 8 day (p < 0.001) groups compared to those with A-β_25–35_ alone and to a level which did not differ significantly from that of controls (24 h p = 0.73; 8 days p = 0.113). No significant effect was observed when E2 was injected two weeks before and two weeks after A-β_25–35_ injection in the 15 day A-β_25–35_ + E_2_ compared with 15 day A-β alone group (p = 0.177 NS).

It called our attention the significant differences observed between the OB vehicle group and A-β_25-35_ plus E_2_ group with the 15 day treatment duration (p = 0.008). This can be interpreted as a possible influence of estrogens in memory.

**Figure 3:**
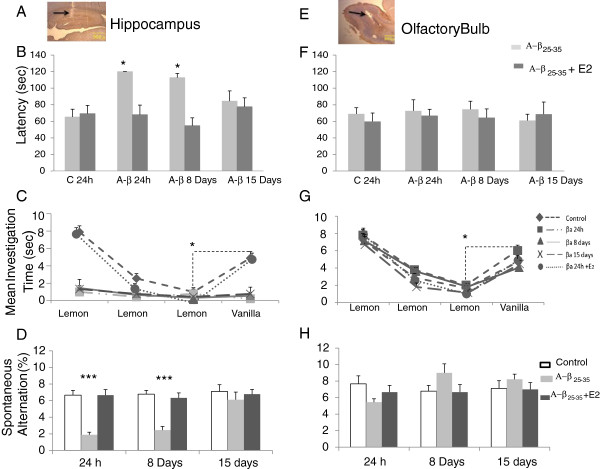
**Behavior test. A** and **E**, Microphotography of HIPPO and OB shows the cannula trajectory (4X). **B** and **F**, Graphs show the latency to find the piece of buried chocolate of ovariectomized animals tested 24 h, 8 and 15 days after HIPPO **(B)** or OB injection **(F)**. A-β_25-35_ injection alone (grey columns). Treatment with E_2_ (black columns). C and G, Graph shows mean investigation time(s) invested by ovariectomized animals to habituate/dishabituate to lemon and vanilla odors, experimental groups tested at 24 h, 8 and 15 days after A-β injection in HIPPO and a group received E_2_ + A-β **(C**, graph**)** or OB **(G**, graph**)**. **D** and **H**, Graphs show percentage of spontaneous alternation triplets in a T-maze of same experimental (grey columns, A-β groups, black columns A-β + E2 treatment) and control groups, white columns. *P < 0.05, ***P < 0.001.

After A-β_25–35_ injection in HIPP, the lesion provoked by the cannula in the CA1 region as well as in the OB can easily be seen. Figure 3A shows a microphotograph of a typical cannula placement in the HIPP and Figure 3E in OB.

Figure 3B and F show mean latencies to locate buried chocolate in the A-β_25-35_ injected HIPP and OB groups and the ones which received additional E_2_. A three-way ANOVA revealed main effects of treatment (F_2, 89_ = 10.25, p < 0.0001), treatment duration (F_2, 89_ = 4.90, p < 0.01) and brain region (F_1, 89_ = 54.3, p < 0.0001). There was also significant interaction between treatment and brain region (F_2,89_ = 6.55, p = 0.002) and between treatment duration and structure (F_2,89_ = 4.9, p < 0.01) showing that the A-β_25-35_ treatments, and their duration, had different effects in the HIPP and in OB. Post-hoc comparisons revealed significant increases in latency in the 24 h, 8 days HIPP A-β_25-35_ groups compared to those of vehicle injected controls or the A-β_25-35_ groups treated with E_2_ (p < 0.05 in all cases) but no differences between the three A-β_25-35_ treatment durations. Indeed, none of the animals were able to find the chocolate within the 120 s test duration, while all the animals in the control group succeeded well within this time. On the other hand, no differences were found between A-β_25–35_ injections from A-β_25–35_ + E2 injection in the olfactory bulb.

Figures 3C and G show the mean investigation times in the habituation-dishabituation odor discrimination paradigm in experimental and control HIPP and OB groups. They were recorded in tests at 24 h, 8 or 15 days after A-β_25-35_ or control injections and for 24 h A-β_25-35_ treatment preceded by two weeks of E_2_. A 4-factor ANOVA with treatment (treatment duration, trials and brain region as factors) revealed significant main effects of treatment (F_2, 161_ = 192.17, p < 0.0001), trial (F_3, 647_ = 395.3, p < 0.0001) and brain region (F_1, 647_ = 45.0, p < 0.0001) but not of treatment duration (F_2, 647_ = 0.93, p = 0.396). There were also significant interactions between treatment and brain region (F_2, 647_ = 54.97, p < 0.0001), treatment and trial (F_6, 647_ = 33.03, p < 0.0001) and between trial and brain region (F_3, 647_ = 2.763, p = 0.041) and also for treatment x trial x brain region (F_6, 647_ = 11.11, p < 0.0001). In general, these show that the A-β_25-35_ treatment only had a significant effect on investigation times across trials in the HIPP compared with those on the OB. Post-hoc analysis revealed that both HIPP and OB (trial 1 p < 0.001 vs. trials 2 and 3 in both cases) control groups showed a clear habituation to the lemon odor test across the three trials and a clear dishabituation (trial 4 vs. trial 3, p < 0.001 in both cases) response was obtained after presentation of a different odor on trial 4 (vanilla). For the HIPP experimental groups tested 24 h, 8 or 15 days after A-β injection, no significant habituation was observed (p > 0.05 in all cases). However, the group that received E_2_ pre-treatment before the A-β_25-35_ injection did not differ significantly from the control groups (p > 0.05 in all cases) while it did from 24 h, 8 and 15 day treatment groups (P < 0.0001 in all cases). The treated A-β OB groups showed a pattern of habituation/dishabituation across trials that did not differ from that of controls (p < 0.05 in all cases).

Figures 3D and H show the effects of HIPP and OB A-β_25-35_ and E_2_ treatments on spontaneous alternation behavior. There were significant main effects of treatment (F_2, 143_ = 15.4, p < 0.0001), treatment duration (F_2, 143_ = 5.63, p = 0.005) and brain region (F_1, 143_ = 8.13, p = 0.005). Significant interactions were found between treatment and brain region (F_2,143_ = 25.42, p < 0.0001) and treatment duration and brain region (F_2,143_ = 10.68, p < 0.0001) indicating that HIPP treatment effects were greater than those for OB treatment at 24 h and 8 day time points. There was also a significant interaction between treatment and treatment duration (F_2,143_ = 18.35, p < 0.0001) indicating again that treatment effects were only at the 24 h and 8 day time points. Post-hoc tests revealed that A-β injection in the HIPP impaired spontaneous alternation behavior at the 24 h and 8 day time points compared with that of the control group and the group pre-treated with E_2_ (p < 0.0001 in all cases). The 24 h E_2_ pretreated group and 15 day A-β_25-35_ treatment groups did not significantly differ from the control one (p = 0.26 and 0.92, respectively).

**Figure 4:**
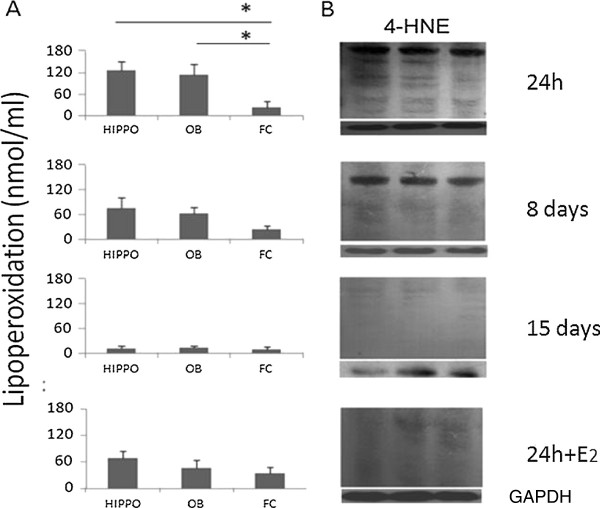
**Effect of A-β injection in HIPPO. A**. Effect of A-β_25-35_ injection in HIPPO on lipid peroxidation levels in HIPPO, OB and Frontal Cortex (FC) of ovariectomized animals. Mean ± SEM lipid peroxidation levels in the four experimental groups (24 h, 8 and 15 days groups and 24 + E_2_). The lipid peroxidation levels in nanomoles/ml of homogenate HIPPO, OB or FC tissue depicted on the ordinate. Minimal amount of lipoperoxidation was detected 15 days after A-β_25-35_ injection. E_2_ replacement decreases the lipoperoxidation in HIPPO as well as in OB. **B** Western blot technique to measure 4-HNE adduct in Hippocampus reflects the lipoperoxidation in HIPPO, OB and FC in the same experimental groups. The amount of 4-HNE adducts, is related with the presence of peroxides in the structure, the administration of E_2_ decreases the presence of peroxides and the amount of 4-HNE.

**Figure 5:**
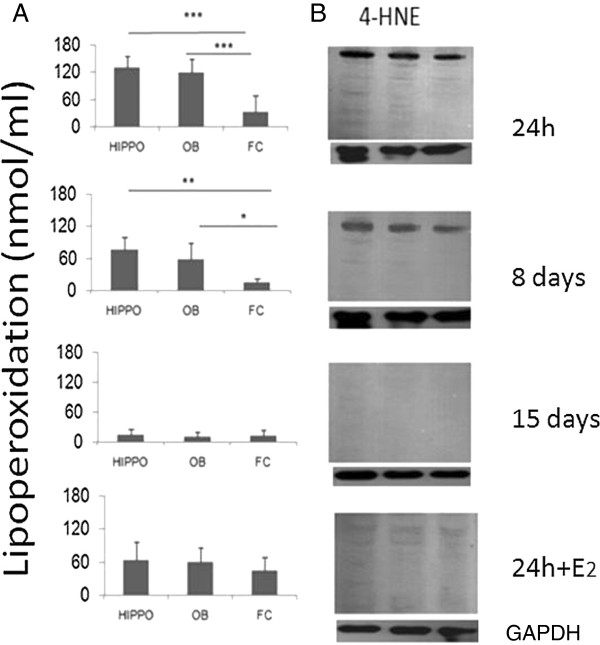
**Effect of A-β injection in OB. A**. Effect of A-β_25-35_ injection in OB on lipid peroxidation levels in HIPPO, OB and Frontal Cortex (FC) of ovariectomized animals. Mean ± SEM lipid peroxidation levels in the four experimental groups (24 h, 8 and 15 days and E_2_ replacement groups). The lipid peroxidation levels in nanomoles/ml of homogenate HIPP, OB or FC tissue depicted on the ordinate. Significant differences were found in 24 h experimental group between HIPPO and OB compared with FC tissue. Minimal amount of lipoperoxidation was detected 15 days after A-β injection in the three structures evaluated. E_2_ decreases the lipoperoxidation in HIPPO as well as in OB. **B**. Western blot technique to measure 4-HNE adduct in OB reflects the lipoperoxidation in HIPPO, OB and FC in the same experimental groups. The amount of 4-HNE adducts is related with the presence of peroxides in the structure, the administration of E_2_ decreases the presence of peroxides and the amount of 4-HNE.

Figures 4A and 5A show that LPO levels were high in both HIPPO and OB by 24 h after A-β_25-35_ injection but not in the frontal cortex (which was used only as a reference structure). A three-way ANOVA revealed that levels of lipoperoxidation (LPO) varied significantly, with main effects of treatment (F_1, 119_ = 20.93, p < 0.0001), treatment duration (F_2, 119_ = 46.23, p < 0.0001) and brain region (F_2, 119_ = 6.13, p = 0.003). There were also significant interactions between treatment and brain region (F_2,119_ = 7.06, p < 0.001) and between treatment duration and brain region (F_4,119_ = 5.05, p < 0.001) indicating that A-β_25-35_ injections produced greater effects in the HIPP and OB than in the frontal cortex. Post-hoc tests showed that the group pre-treated with E_2_ showed significantly lower levels of lipoperoxidation in both HIPP and OB 24 h after A-β_25-35_ injection compared to those of the group treated with A-β_25-35_ alone for 24 h (p < 0.05 in both cases). The Western Blot analyses of lipid peroxidation 4-hydroxy-2-nonenal (4-HNE) protein adduct showed high levels after A-β_25-35_ injection in HIPPO and OB but not in the frontal cortex (Figures 4B and 5B). The A-β_25-35_ HIPP injection group treated with E_2_ showed a reduced presence of 4-HNE (Figure 4B).

**Figure 6:**
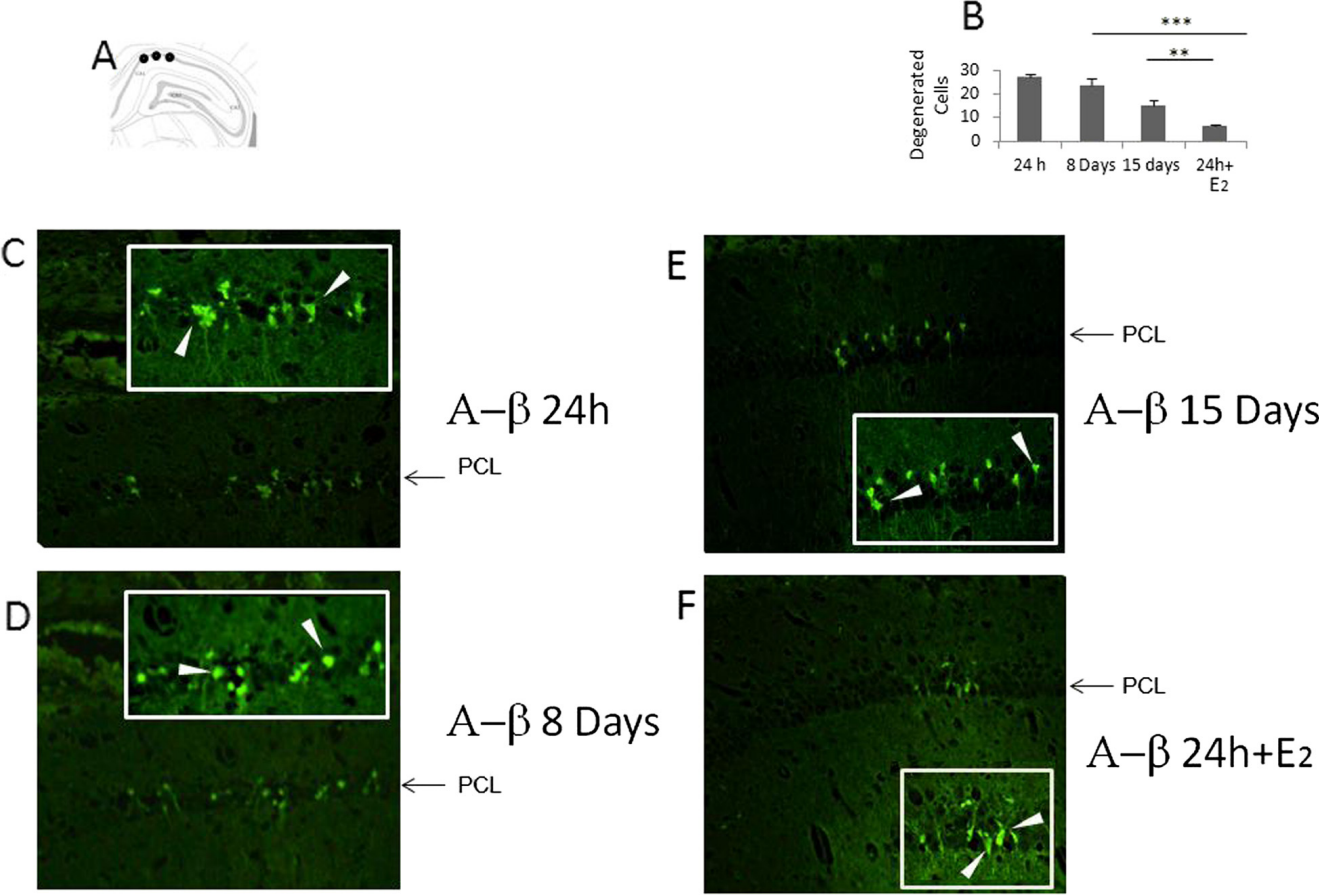
**Injection sites. A**. Scheme of A-β_25-35_ injection sites (Ca1 subfield). **B**. Only Fluorojade positive cells staining were counted. **C**, **D**, **E**. Microphotography’s of HIPPO using Fluorojade staining after A-β_25-35_ injection in HIPPO and sacrificed at 24 h, 8 or 15 days later. Insert shows 40x magnification of Ca1 subfield. **F**, pretreatment two weeks before A-β _25–35_ with E_2_.

Fluoro-Jade staining revealed the presence of degenerating neurons in HIPP at 24 h, 8 days and 15 days after A-β_25-35_ injection (Figures 6C,D,E and F) but not in the OB (data not shown). There was also no evidence for Fluoro-Jade stained degenerating neurons in the HIPP or OB following OB injection of A-β_25-35_. A two-way ANOVA was therefore performed only on the groups with HIPP injections and with treatment and treatment duration as factors. This showed significant main effects of treatment (F_1, 15_ = 18.67, p < 0.001) but not treatment duration (F_2, 15_ = 0.269, p = 0.769). Post-hoc pairwise comparisons revealed a significant difference between the 24 h A-β_25-35_ injected group and the 24 h A-β group pre-treated with E_2_ (p < 0.001). There was also a significant reduction in the number of staining cells in the A-β_25-35_ 15 day group compared with the 24 h one (p < 0.05).

## Discussion

Our results demonstrate that bilateral injections of the A-β_25-35_ fragment in the HIPP of ovariectomized female rats produce marked deficits in olfactory perception and social recognition and spatial memory as shown in Figures 2 and 3. Bilateral injections of the same dose of A-β_25-35_ into the OB did not produce any behavioral impairment. These behavioral effects of HIPP A-_25–35_ β injections were associated with increased LPO and 4-NE, in both HIPP and OB; although only with injections into the HIPP did actual neuronal degeneration occur in the HIPP, as shown in Figure 4. These behavioral and degenerative effects of A-β_25-35_ injection occurred at 24 h and 8 days after treatment although they had largely disappeared by 15 days post injection. It is important to highlight that two weeks pre-treatment before A-β_25-35_ injection, with E_2_ or one or two weeks after prevented the occurrence of all perceptual and memory impairments and significantly reduced associated neurodegenerative changes. Thus, E_2_ treatment can play a potent role in protecting the brain from the neurotoxic effects of A-β_25-35_.

A-β is the main constituent of senile plaques found in the aging brain and has been extensively linked with disturbances of learning and memory processing characteristics of aging-associated disorders, such as AD [1,41]. It is also known that aggregation of the amyloid peptides is responsible for neurotoxicity [20,42,43].

Up to date, there is no data regarding the formation of plaques in A-β_25–35_ injection models. Which was neither observed in our model in any of the time points being assessed (24 h, 8 and 15 days). The injections of A-β_25-35_ did not produce neurodegenerative changes restricted to the region of the injection. At this point, we are unsure how the A-β_25-35_ spread from the HIPP to OB and vice versa despite simple transport within the cerebroventricular system seems unlikely due to the absence of effects in the frontal cortex. Instead, a more likely explanation is transport along migratory routes between the two structures. Both HIPP and OB are sites of neurogenesis within the brain but also where cells migrate from the sub-ventricular zone into both regions. [44]. Stem cells applied intranasally have also been shown to track from olfactory regions into the HIPP [45] so our findings may suggest a mechanism where A-β formation occurring within the OB can rapidly move into the HIPP and vice versa.

Our finding that both olfactory perception and social recognition memories were impaired following A-β_25-35_ injection into the HIPP was also unexpected as a previous research work suggested a role for the HIPP in social recognition memory [46] and other forms of olfactory memory [47-49] but not in olfactory perception *per se*. Possibly, the profound olfactory perception deficits we observed may have been caused by the spread of A-β from the HIPP to the OB, although we did not find similar deficits following direct injection of the same A-β_25-35_ dose into the OB despite similar levels of lipoperoxidation. However, as a result of the olfactory perception deficits, we obviously cannot conclude that social recognition memory was impaired since this is highly dependent on odor cues [50]. Nevertheless, since deficits in a non-odor dependent spatial memory task spontaneous alternation were also found, we can conclude that the A-β_25-35_ injection into the HIPP impaired both olfactory perception and spatial learning. These data suggest that neurodegeneration in HIPP could explain in part, olfactory impairment found in some neurodegenerative diseases such as Alzheimer’s.

Our findings show that oxidative stress due to A-β_25-35_ injection failed to produce actual neurodegeneration in the OB which was expected to happen given the effects observed following HIPP injections. However, there is evidence that the pyramidal neurons of the CA1 HIPP subfield are very sensitive to oxidative stress [51] and so perhaps this may explain why only the HIPP show actual evidence for neurodegenerative cells thus resulting in behavioral changes. Other studies have also reported that A-β_25-35_ can damage the HIPP and impair learning and short-term memory [15,52,53]. Another one has reported that bilateral injection of A-β_25-35_ into the amygdala of rats induced histopathological changes such as the appearance of reactive astrocytes and neuronal shrinkage, but did not cause any disturbance in spatial learning or in conditioned avoidance learning [54]. Interestingly, in agreement with our observations, spatial memory impairments following intracerebroventricular (i.c.v) injections of A-β_25-35_ have also been reported to be correlated with actual neuronal cell loss in HIPP [53].

LPO is a reliable marker of oxidative stress because it reflects damage to membranes and produces a variety of damaging reactive oxidizing species associated with cell death [55]. For instance, oxidative stress caused by environmental *stimuli* is proposed to be involved in brain neuronal death in many neurodegenerative disorders such as Alzheimer’s and Parkinson’s diseases [56].

Previous evidence from our laboratory has shown that ozone inhalation causes oxidative stress in a number of different brain regions in rats [57,58] and in this paper, we show that A-β_25-35_ injection in the HIPP increases LPO in it as well as in the OB compared with control groups. It is well known that HIPP is one of the key sites vulnerable to neurotoxicity *in vivo* and in relation to AD [52,59].

Our experiments showed that both behavioral and neurodegenerative impairments induced by A-β_25-35_ injections were transient with changes either fading or disappearing by 15 days post-injection. To the best of our knowledge, this ability of the brain to largely recover from the neurotoxic effects of A-β_25-35_ injections has not been reported, with most studies focusing on single time points [15,27,52].

For instance, in the hippocampus, there are reports that CA1 region neurons are more susceptible to oxidative stress impairment than CA2 or CA3 neurons [60]. The aforementioned statement means that even though similar oxidative levels are produced by the A-β_25-35_ injection in both sites HIPP and OB, it results in a neuronal degeneration in only the CA1 region of the hippocampus but not in the that of the olfactory bulb where the olfactory behavior remains intact even after being the A-β_25-35_ injected directly in the bulb. In fact, in order to produce an olfactory behavior impairment injecting the A-β_25-35_ in the OB, we need to administer a double dosage than that in HIPP (4 μl), (data not shown), which evidences the susceptible difference to oxidative stress between hippocampus and olfactory bulb neurons.

A-β_25-35_ injection in the hippocampus produces a fluctuation in the spatial behavior [15,61]. In our model, we found that there are also fluctuations in the rat’s olfactory behavior; these are observed in the first few days after A-β_25-35_ injection as Figures 2 and 3 show. However, a recovery of the olfactory behavior is observed afterwards.

It has been reported that cell neurogenesis in the subventricular area and its migration to the lesion area may partly explain this recovery [62]. Our injection model shows that the affected neurons are those found in an adjacent A-β_25-35_ injected area, no bigger than 600 microns, thus the impairment does not invade other areas of the hippocampus keeping the rest of the structure’s functions intact.

Some studies have reported memory impairments following i.c.v A-β_25-35_ administration after periods around or in excess of 15 days [15]. It is possible, therefore, that the brain’s capacity to compensate following A-β treatment may be increased when localized injections in the HIPP or OB are used as opposed to more global i.c.v administration. There is continuous cell migration from the subventricular and subgranular zones of the HIPP to the OB and to the HIPP itself following damage [59]. Thus, possibly, cell migration from the subventricular zone to the OB together with neurogenesis within the OB contributed to both functional and neurodegenerative recovery by 15 days after HIPP A-β_25-35_ injections and E_2_ treatment.

The A-β_25-35_ induced neurodegeneration is traceable by means of a Fluoro-Jade C technique which is positive from 24 hours after injection. This technique mainly stains the neurons in degeneration process [63]. This degeneration will result in cell death and the neuronal remains will eventually vanish together with the astrogliosis and inflammatory reaction. As the Fluoro-Jade C is mainly used to signal the cells in degeneration process, the intensity of the signal gathered at day 15 is lesser than that obtained at 24 hours or 8 days later, there are scarcely left few neuronal remains, thus, less fluorescence. When we assess hippocampus cuts stained with eosin and hematoxylin after 15 days, we can observe the absence of pyramidal neurons in the injected area.

Neuroprotective actions of estradiol have been shown in a number of different contexts [29,32]. The 17 β-estradiol dosage used in this research work has shown to have antioxidant effects in other models such as the exposure to ozone [57,58]. In the current study, the protective effects we observed following a two week pre-treatment and a one or two weeks after E_2_ in ovariectomized rats were clearly very strong, with a complete absence of any olfactory perception or olfactory learning or spatial learning deficits. While, following the E_2_ treatment, there was still some evidence for increased lipoperoxidation and neurodegenerative changes at 24 h after A-β_25-35_ treatment in either HIPP or OB; this was significantly lower compared with that of A-β_25-35_ treatment alone. There is a significant decrease in the lipoperoxidation levels after A-β_25–35_ injection in the group with estradiol supplement, while in the groups without it the oxidative stress levels were higher. It can be observed that the dosage used (25 mg/kg) has an antioxidant effect which is reflected in a lower neuronal degeneration which is related to a lesser intensity of the Fluoro-Jade stain.

We have previously shown that similar E_2_ treatment to ovariectomized rats protects against ozone-induced olfactory memory deficits and lipoperoxidation in the olfactory system [58]. Here, we have extended these findings to include protection against the neurodegenerative and behavioral effects of A-β.

We deliberately chose to use an ovariectomy model in order to demonstrate potential neuroprotective effects of E_2_ treatment since it reflects similar hormonal changes that occur in women following menopause. While the incidence of AD is significantly higher in women than in men, clear evidence that post-menopausal reductions in estrogens contribute to this as opposed to greater longevity has yet to be produced [64-66], despite early influential studies suggesting otherwise [30,31]. It does, however, seem that there may be a particular period of vulnerability in the early stages of menopause and there is still considerable interest in establishing potential therapeutic efficacy of estrogen treatment [64]. At this stage, studies in rodents have reported that brain estrogens deficiency can accelerate A-β plaque formation in a transgenic mouse model of AD [67]. It also seems to be that both estrogen α and β-receptors may contribute to increases and decreases respectively in hippocampal apolipo protein E expression [68]. Furthermore, the potential neuroprotective mechanism whereby estrogen is acting to reduce A-β may be due to reductions in oxidative stress via the mitochondria. Clearly, we still need further evidence to support both estrogen interactions with A-β injection as well as its potential for therapeutic use in AD.

## Conclusions

In summary, our results have demonstrated significant impairments of olfactory perception and spatial memory function 24 h and 8 day following injection of A-β_25–35_ in the HIPP, but not in the OB of ovariectomized rats. These behavioral changes were associated with evidence of high levels of lipoperoxidation in both HIPP and OB and the presence of degenerating neurons in HIPP. A two-week pre-treatment or one or two weeks after with E_2_ in ovariectomized rats completely prevented the occurrence of behavioral impairments and markedly reduced neurodegenerative changes 24 h after A-β_25-35_ injection into the HIPP. These results further suggest an important neuroprotective role for estrogens against A-β_25-35_ induced neurotoxic damage with potential relevance to treatment of AD, particularly in the context of post-menopausal women.

## Abbreviations

AD: Alzheimer’s disease; A-β: Amyloid beta; i.c.v.: Intracerebroventricular; APP: Amyloid precursor protein; ROS: Reactive oxygen species; E2: 17β estradiol; i.p: Intra peritoneal; IEI: Inter exposure interval; HIPP: Hippocampus; OB: Olfactory bulb; s.c.: Subcutaneous; LPO: Lipoperoxidation.

## Competing interests

The authors declare that they have no competing interests.

## Authors’ contributions

CBM participated in the design of the study, experimental procedure and writing of the manuscript. SRA participated in the design of the study an analysis of the results. KMK participated in the analysis of the data and the writing of the manuscript. RGG participated in the design of the study, analysis of the data, corrected the manuscript and guidance through the project. All the authors read and approved the final manuscript.
